# Comparison of reverse torque values of abutment screws with the application of oil-based and water-based antibacterial agents

**DOI:** 10.34172/joddd.2022.038

**Published:** 2022-12-30

**Authors:** Amin Nourizadeh, Elnaz Shafiee, Azin Khorramdel, Seyed Amin Mousavi, Mehran Rahbar

**Affiliations:** ^1^Department of Prosthodontics, Faculty of Dentistry, Tabriz University of Medical Science, Tabriz, Iran; ^2^Department of Prosthodontics, Faculty of Dentistry, Islamic Azad University, Tabriz, Iran; ^3^Department of Periodontics, Faculty of Dentistry, Islamic Azad University, Tabriz, Iran; ^4^DDS, Private Practice, Tabriz, Iran

**Keywords:** Abutment, Chlorhexidine, Implant, Reverse torque, Tetracycline

## Abstract

**Background.:**

Using antibacterial agents to remove the foul odor of the implant cavity and prevent peri-implantitis can affect the detorque values and lead to the loosening of the abutment screw. This study investigated the effects of tetracycline and chlorhexidine gel on detorque values.

**Methods.:**

This in vitro study was carried out on three groups of five implants. Group G1 was the control group, and no material was applied to the implant cavity. In group G2, the implant cavity was first filled with artificial saliva and then with chlorhexidine gel. In group G3, the implant cavity was first filled with artificial saliva and then with tetracycline. The abutments were tightened with 25 N/cm^2^ and then loosened. Finally, the detorque values were calculated.

**Results.:**

The highest detorque values were recorded in group G1. Group G3 showed the lowest detorque values. ANOVA showed significant differences in mean detorque values (*P*<0.05) between the three groups.

**Conclusion.:**

According to this study, applying antibacterial agents decreased the detorque values and increased the risk of screw loosening. The reduction of detorque values was more pronounced with the oil-based antibacterial agent (tetracycline).

## Introduction

Dental implants have become one of the most widely used treatment modalities in modern dentistry, and advances in implantology have significantly improved the quality of treatments offered to patients.^1,2^ According to recent studies, the success rate of implant treatments is 97‒99%, which has led to the increasing use of dental implants.^2^ However, mechanical and biological problems that lead to implant failure continue to be reported.^3,4^ Mechanical complications include screw fractures, implant fractures, and restoration fractures.^5^ Biological complications include excessive force on the bone, bone resorption, and microflora diffusion in microgaps between the implant and abutment.^6^

Among the mechanical complications, screw loosening is the most important regularly reported complication. It can cause micromotion and other problems, such as inflammation of the soft tissue around the implant (peri-implantitis) and breakage of the screw.^6^ The incidence of screw loosening varies depending on the type of restoration, and the highest rate was observed with single crowns.^7^ In other studies, the screw loosening rate was around 12.7% for single crowns^8-10^ and 6.7% for fixed partial dentures.^9-11^ Inadequate preload, screw shape, cantilever structures, occlusion scheme or incorrect crown anatomy, bone remodeling, and bruxism are some of the reasons for screw loosening.^12^

The colonization of the microflora and the presence of microorganisms in the microgap of the implant and abutment cause an unpleasant odor for the patient and the restorative dentist when the healing abutment is opened during prosthetic appointments.^13^ Implant systems with screw retention modalities have been used with high success rates for years. In this construction, supra structures are connected to the implant body with a titanium screw.^14^ The presence of inflammation at the implant‒abutment level affects the implant’s durability and jeopardizes its survival.^15-17^ Microleakage at the implant‒abutment connection is the most important cause of the inflammatory reaction around the implant.^18^ Microleakage leads to bacterial colonization around the implant‒abutment complex, which leads to the onset of the pathophysiological process of bone loss followed by implant loss.^8-10^

The penetration of microorganisms into the microgap can lead to inflammation in the soft tissue around the implant (peri-implantitis) and mucositis around the implant (peri-implant mucositis).^19^ In addition, bacterial infection can lead to bone loss and disrupt the osseointegration process in the postoperative repair phase.^20^ The penetration of microorganisms can occur during the opening and closing of the abutment or after loading the implant.^21^

To prevent these possible side effects, the use of antibacterial agents is recommended. According to a study by Micarelli et al,^22^ using an antimicrobial agent such as chlorhexidine on the implant cavity before covering the screw or healing abutment may reduce the accumulation of bacteria and the leakage of bacteria and toxins into the implant cavity. Another study showed that applying 1% chlorhexidine gel to the implant cavity before the abutment was placed over six months led to a significant reduction in bacterial colonization in the implant cavity.^23^ The presence of these antibacterial agents can cause slippery surfaces, ultimately affecting the torque and post-rotation of the abutment screw. In one study, applying 0.2% chlorhexidine gel to the implant cavity reduced the amount of detorque and preload and increased the risk of screw loosening.^24^

Nevertheless, the drying out of the implant cavity can reduce the tension when tightening the abutment screw, increasing the likelihood of screw loosening, loosening of the prosthesis, screw breakage, and peri-implantitis.^10^ According to other studies, the presence of saliva has no significant effect on the level of torque and detorque.^25^ Another study found that placing zirconia abutments in the implant cavity in the presence of saliva had a greater degree of detorque than placing abutments in the dry implant cavity.^26^ However, the effect of other oil- and water-based antibacterial agents on torque and detorque values has not been evaluated.

 Considering the limited studies on the effects of various antibacterial agents on the torque and detorque values of abutment screws, different results have been reported on changes in the detorque values, and there are contradictions regarding the change in the detorque values. Therefore, this study measured and compared the detorque values of abutment screws with two types of oil- and water-based antibacterial agents.

## Methods

According to a study by Jo,^19^ the means ± SD of the abutment screw detorque in the control and chlorhexidine groups were considered 19 ± 0.43 and 17.28 ± 0.39, respectively, with α = 0.05 and β = 0.80. Therefore, the sample size was calculated at four implants in each group. However, to increase the study’s validity, five implants were included in each group, totaling 15 implants.

**Figure 1:**
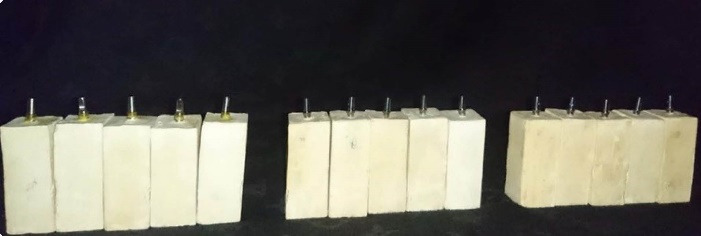
Dividing mounted fixtures into three groups of five

**Figure 2:**
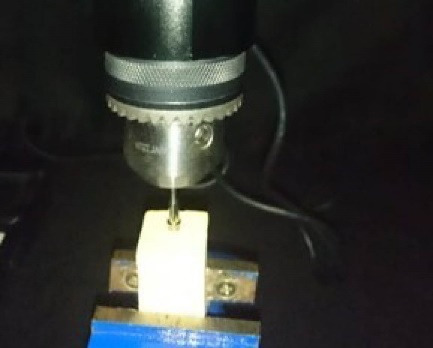
Mounting fixtures via surveyors

Fifteen DIO UF cylindrical fixtures (Dio Implants, Seoul, Korea) with a diameter of 41 mm and a length of 11.5 mm were divided into three groups of five (Figure 1). In addition, 15 DIO UF abutments (Dio Implants, Seoul, Korea) with a diameter of 4.5 mm and a GH (gingival height) of 2 mm were selected. Implant fixtures were mounted into plaster blocks using Die stone (gypsum type IV) (Velmix, Kerrdental, United Kingdom) via surveyors (Figure 2). First, the implants were placed in a container filled with artificial saliva (Aquoral, 0.4% hyaluronic acid).

In group G1, the implant cavity was only soaked with artificial saliva (Aquoral, 0.4% hyaluronic acid). After one week, the implants of this group were retrieved from the container of artificial saliva, their cover screw was removed, and the implant cavity was filled with artificial saliva. Then the healing abutment was tightened to 15 N/cm^2^ and returned to the container. After one week, the implants were retrieved from the saliva container, the healing abutments were removed, and the abutments were replaced and tightened with a torque of 25 N/cm^2^.

In group G2, the implant cavity was first soaked with artificial saliva and then with 0.2% chlorhexidine (TePe chlorhexidine gingival gel, 0.2%). After one week, the implants in this group were retrieved from the container, their cover screw was removed, and the implant cavity was filled with 0.2% chlorhexidine. Then the healing abutment was tightened to 15 N/cm^2^ and returned to the container. After one week, the implants were retrieved from the saliva container, the healing abutment was removed, and the abutment was reinserted and tightened with a torque of 25 N/cm^2^.

**Figure 3:**
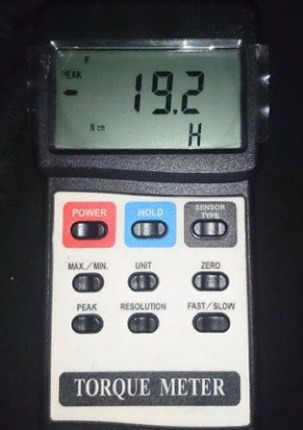
Controlling the load via a digital torque meter

In group G3, the implant cavity was first impregnated with artificial saliva and then with 3% tetracycline (Aerotex, tetracycline topical ointment, 3%). The implant cavity was filled with 3% tetracycline, and then the healing abutment was tightened with 15 N/cm^2^ and returned to the saliva container. After one week, the implants were retrieved from the saliva container, the healing abutment was removed, and the abutment was reinserted and tightened with a torque of 25 N/cm^2^. Due to the influence of the screw tightening speed on the torque value, the screws were closed with a digital torque meter (Digital Torque Meter, TQ-8800, Lutron, Taiwan). The load applied by the digital torque meter was controlled, and the exact torque required to tighten the screw was displayed on the device monitor (Figure 3). Due to the intact connection between the abutment and the implant cavity, no mechanical tests were carried out before applying the detorque force. The abutments were soaked in synthetic saliva, 0.2% chlorhexidine, and 3% tetracycline in groups G1, G2, and G3, respectively, before they were tightened with a torque of 25 N/cm^2^. The time interval between tightening and loosening the screw was 15 minutes. Each time the screw abutments were loosened and tightened with a torque of 25 N/cm^2^, the detorque values were recorded on the device monitor.

To compare the detorque values between the study groups, analysis of variance, or its non-parametric equivalent, the Kruskal-Wallis test, was used. SPSS 17 was used for data analysis. *P* < 0.05 was considered statistically significant.

## Results

Table 1 presents the mean ± SD values of the reversal torques of the abutment screws in the study samples. The highest amount of reverse torque was recorded in artificial saliva (23.37 ± 1.09), with 20.02 ± 1.81 in the chlorhexidine group. The lowest reverse torque value (16.25 ± 1.67) was recorded in the tetracycline group.

**Table 1:** Descriptive statistics of abutment screw’s reverse torque values in the study samples

**Variable**	**Mean±SD**
Artificial saliva	23.37 ± 1.09
Tetracycline	16.25 ± 1.81
Chlorhexidine	20.02 ± 1.67

 The distribution of the variables in each group was analyzed by the Kolmogorov-Smirnov test, and because of their normal distribution, a parametric test, such as ANOVA, was used to compare them. Post hoc Tukey tests were used for two-by-two comparisons of the groups.

According to the Tukey test results in Table 2, the difference in reverse torque values between saliva and tetracycline groups was significant (*P* < 0.05). In addition, there was a significant difference between the salivary and chlorhexidine groups and between the tetracycline and chlorhexidine groups (*P* < 0.05).

**Table 2:** Intergroup analysis in the study samples

	**Group**	**Group**	***P *** **value**
Reverse torque	Artificial saliva	Tetracycline	0.000*
	Artificial saliva	Chlorhexidine	0.001*
	Tetracycline	Chlorhexidine	0.000*

*P value < 0.05 was considered significant.

## Discussion

One of the problems with implant prostheses is the loosening of the screw that connects different parts. Unstable and loose prosthetic screws can lead to more serious complications such as screw fracture, prosthesis loosening, or implant failure.^27^ Incorrect torque when tightening the screw is an influential factor in screw loosening and breakage and other technical problems with implant systems.^28,29^ One of the causes of unintentional loosening of screws is insufficient torque,^30^ and one of the causes of screw fractures is excessive torque.^31^

When exerted on natural teeth, functional and parafunctional forces create a physiological adjustment in the periodontal tissue, but occlusal trauma can result when this force is greater than adaptive capacities. Unlike natural teeth, excessive occlusal forces due to the lack of periodontal ligamentaround the implant fixtures can cause mechanical problems such as screw loosening, screw failure, and even fixture failure. The most important and common implant problems include implant failure, loosening, infection, inflammation, bone and tissue failure, damage to adjacent structures, etc.^32^

Controlling inflammation around dental implants is critical to reducing the rate of bone resorption in the crestal region, the health of the surrounding soft tissue, and increasing the efficiency and life of dental implants.^33^ Inflammatory processes around implants are relatively similar to those around natural teeth, except that infections around implants cause more destruction, mainly due to the absence of periodontal ligaments.^34^ The gap between the two components causes bacterial proliferation, inflammation, and bone loss around the implant.^35,36^ Bacterial biofilms around the implant‒abutment complex affect the biological width, compromising the bone margin. Finally, the soft tissue margin recedes, which affects the aesthetic outcome.^37,38^

A major challenge is preventing bacterial proliferation at the implant‒abutment connection to minimize inflammatory reactions and maximize bone stability in the crestal area.^18^ The current study examined the effect of two different antibacterial agents used to control microbial growth and reverse torque values of the abutment screw. In this study, the reverse torque with artificial saliva was 23.37 N/cm^2^, which was higher than the other two groups of tetracycline and chlorhexidine. ANOVA and Tukey tests revealed that this difference was significant.

Not using antibacterial agents increases the accumulation of bacteria and leakage into implant cavities,^22^ leading to inflammation at the implant‒abutment connection and reducing implant durability.^15-17^ Microleakage at the implant‒abutment connection is the most important cause of the inflammatory reaction around the implant.^18^ Microleakage causes bacterial colonization around the implant‒abutment complex, leading to the onset of the pathophysiological process of bone loss, followed by implant loss.^39,40^

When chlorhexidine was used in the second group of this study, the reverse torque reached 20.02 N/cm^2^. Chlorhexidine reduces the accumulation of bacteria, toxins, and bacterial leakage into the implant cavity,^22^ and according to the current study, it reduces the level of the detorque values. These antibacterial agents in the implant cavity can lead to slippery surfaces and reduce the detorque values of abutment screws.

Asli et al^24^ reported that 0.2% chlorhexidine gel on the implant cavity reduced the detorque and preload values and increased the risk of screw loosening, consistent with the present study. Paolantonio et al^23^ showed that using 1% chlorhexidine gel in the implant cavity before placing the abutment significantly decreased bacterial colonization in the implant cavity over six months. The detorque value in the chlorhexidine group was similar to that in a study by Micarelli et al.^22^ They also stated that contamination reduced the level of detorque. Since contamination was unavoidable in a laboratory process, they suggested using plasma argon cleaners to decontaminate the screw‒abutment complex.

Tetracycline decreased the detorque value in the third group in this study, which was greater than in the second group. The detorque value was 16.25 N/cm^2^, which was significantly lower than in the second group. Park et al^13^ also showed that the detorque value in the tetracycline group decreased due to the increased slipperiness. The greater reduction in this compared to the chlorhexidine group was attributed to the nature of the antibacterial agents. Chlorhexidine is water-based, and tetracycline is oil-based, and the lubricity of tetracycline is higher than that of chlorhexidine. Therefore, the reduction in detorque values was greater with tetracycline. The presence of a lubricant and its type in the screw‒abutment complex can reduce the coefficient of friction, depending on the type of lubricant.^34^ Since both chlorhexidine^22^ and tetracycline reduced reverse torque values in this study, these two substances are not considered suitable lubricants.

 The present study was conducted by taking into account some hypotheses and had some limitations. To improve and expand the topic, the following should be investigated: the bacterial accumulation around the abutment, the effect of antibacterial lubricants, and the effect of various impurities and cleaners on detorque values.

## Conclusion

 According to the present study and other studies, using antibacterial agents reduces the detorque values of abutment screws. The reduction in detorque with oil-based antibacterial agents is more than that with water-based ones.

## Acknowledgments

 The authors would like to thank the Prosthodontics Department, Faculty of Dentistry, Tabriz University of Medical Sciences, for their cooperation.

## Funding

 No funding was requested for this study.

## Ethics Approval

 The study protocol was approved by the Ethics Committee of Tabriz University of Medical Sciences under the code IR.TBZMED.VCR.REC.1398.438.

## Competing Interests

 The authors declare no competing interests with regard to the authorship and/or publication of this article.
